# Exploring the Cognitive Health of LGBTQIA+ Older Adults from The Stonewall Generations Study

**DOI:** 10.1002/alz.091144

**Published:** 2025-01-09

**Authors:** Jason D. Flatt, Brittany Klenczar, Jalal Uddin, Max OHala, Sarah Benson, Andrew Rosenblatt, Shehroo Pudumjee

**Affiliations:** ^1^ University of Nevada Las Vegas School of Public Health, Las Vegas, NV USA; ^2^ Jona Goldrich Center for Alzheimer’s and Memory Disorders, Cedars‐Sinai, Los Angeles, CA USA; ^3^ Cleveland Clinic Lou Ruvo Brain Health Center, Las Vegas, NV USA

## Abstract

**Background:**

Over 2.5 million LGBTQIA+ (lesbian, gay, bisexual, transgender, queer, intersex, asexual or another sexual and/or gender minority identity) adults in the U.S. are aged 50 or older. Past studies have found that LGBTQIA+ older adults report worsening memory and thinking skills more often than non‐LGBTQIA+ older adults. The purpose of this study was to explore the risk and protective factors associated with the cognitive health of LGBTQIA+ older adults.

**Methods:**

We utilized tailored recruitment and engagement strategies to recruit a community‐based sample (n = 67) of racial/ethnically diverse LGBTQIA+ older adults aged 50+ from the U.S. To examine cognitive health, we used the Telephone Interview for Cognitive Status (TICS), with scores ranging from 0‐41. Descriptive statistics were used to summarize demographic characteristics and TICS scores, and we used bivariate analyses to explore potential risk and protective factors associated with cognitive health.

**Results:**

Mean age was 64.5 with range of 50 to 87 years. About 6% had high school degree or less and 23% reported an annual income of $30,000 or less. In terms of race/ethnicity, 85% identified as White, 10% Black, 6% American Indian/Alaska Native, and 10% Hispanic/Latino. For gender identity, 36% identified as women, 39% men, 18% transgender men/women, 12% another gender identity, and one person identified as intersex. For sexual orientation, 43% identified as gay, 36% lesbian, 12% bisexual, 10% queer, 9% heterosexual, 8% asexual, and 5% another identity. TICS scores ranged from 16 to 41 with a mean of 28.05 (SD = 4.92), with 16% falling into the mildly impaired and 9% in the moderately‐to‐severely impaired ranges. A lower TICS score was associated with lower education, functional impairment, past LGBTQIA+ related discrimination (minority stress), and PTSD‐related symptoms (p‐values <0.05).

**Conclusions:**

Our current findings highlight the need for additional research on risk and protective factors for the cognitive health of diverse LGBTQIA+ older adults. Understanding the risk of cognitive impairment among LGBTQIA+ older adults is critical to defining the burden of Alzheimer’s disease and developing targeted prevention strategies for these understudied populations.

